# A Sustainable Approach to Fabricating Ag Nanoparticles/PVA Hybrid Nanofiber and Its Catalytic Activity

**DOI:** 10.3390/nano5021124

**Published:** 2015-06-23

**Authors:** Yongde Meng

**Affiliations:** Department of Chemistry, Hanshan Normal University, Chaozhou 521041, China; E-Mail: myd@hstc.edu.cn; Tel.: +86-768-231-8680; Fax: +86-768-231-8681

**Keywords:** nanofiber, catalytic activity

## Abstract

Ag nanoparticles were synthesized by using *Ficus altissima* *Blume* leaf extract as a reducing agent at room temperature. The resulting Ag nanoparticles/PVA mixture was employed to create Ag nanoparticles/PVA (polyvinyl alcohol) hybrid nanofibers via an electrospinning technique. The obtained nanofibers were confirmed by means of UV-Vis spectroscopy, The X-ray diffraction (XRD), Fourier transform infrared (FTIR) spectroscopy, scanning electron microscopy (SEM), transmission electron microscopy (TEM), and then tested to catalyze KBH_4_ reduction of methylene blue (MB). The catalytic results demonstrate that the MB can be reduced completely within 15 min. In addition, the Ag nanoparticles/PVA hybrid nanofibers show reusability for three cycles with no obvious losses in degradation ratio of the MB.

## 1. Introduction

In recent years, synthesis of nanofibers composed of Ag nanoparticles and polyvinyl alcohol (PVA) via an ecletrospinning technique has attracted increasing attention due to their unique physical and chemical properties. The Ag nanoparticles/PVA hybrid nanofibers show various applications as sensors [1], antimicrobial materials [2], wound dressings [3], surface-enhanced Raman scattering (SERS) detectors [4], catalytic agents [5] and dye-sensitized solar cells [6].

So far, several methods have been developed to fabricate the Ag nanoparticles/PVA hybrid nanofibers. These methods can be classified into two general groups. In one group, the Ag nanoparticles are synthesized using chemical reduction methods. The obtained Ag nanoparticles are added to the PVA dissolved in water and electrospun [7]. In the other group, silver salt is added to the PVA solution to form mixture. The resulting mixture is electrospun into nanofibers. The prepared nanofibers containing silver ions are subjected to physical methods to reducing the silver ions, such as thermal treatment [8], UV irradiation [9] microwave irradiation [10] and so on. In general, these methods to fabricating the Ag/PVA hybrid nanofibers are expensive and hazardous to the environment due to involvement of toxic reducing agents, high temperature, harmful UV light or microwave radiation during the preparations. Hence, sustainable approaches for the preparation of Ag nanoparticles/PVA hybrid nanofibers are desired because of increasing environmental concerns.

*Ficus altissima Blume* is a common plant in Southeast Asia [11]. It is also commonly found on authors’ campus. Among various plants on campus, the *Ficus altissima Blume* leaves are green all the year round, so it is much convenient for researchers to collect the fresh leaves for the present study. In addition, it is also environmentally friendly to synthesize Ag nanoparticles using the *Ficus altissima Blume* leaf extract as a reducing agent at room temperature. Without extracting Ag nanoparticles subquently, the prepared reaction medium is mixed with PVA to form mixture. Then, the mixture is electrospun to obtain the Ag nanoparticles/PVA hybrid nanofibers.

As a cationic dye, MB is often used in the textile. It can be found in wastewater. Due to its toxicity to aquatic creatures and harm to human beings, removal of the MB from aqueous solution is environmentally important. Therefore, the MB is used as a model sample to evaluate catalytic activity of the formed composite nanofibers.

KBH_4_ (potassium borohydride) is known as a reducing agent. It is often used to reduce organic or dyes. Due to its lower cost than NaBH_4_ (sodium borohydride), it is selected to degrade the MB in the presence of the Ag nanoparticles/PVA hybrid nanofibers in the present study.

## 2. Results and Discussion

**Figure 1:**
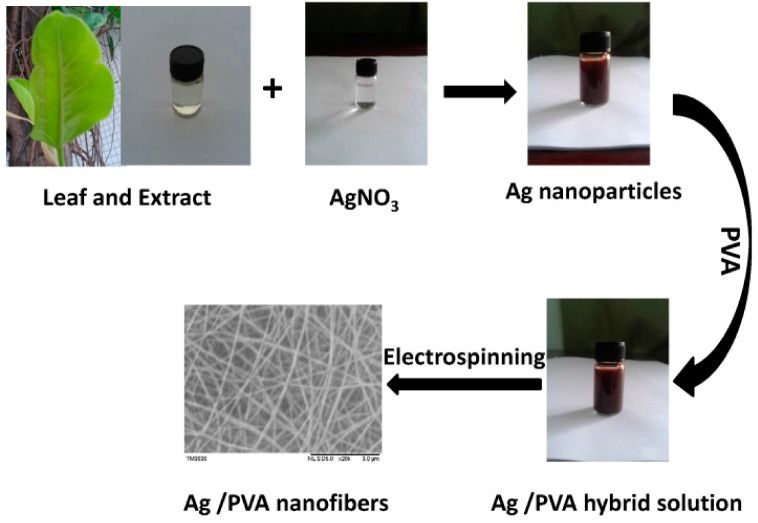
Schematic diagram of process for preparing Ag nanoparticles/PVA (polyvinyl alcohol) nanofibers.

Figure 1 shows procedure for preparation of the Ag nanoparticles/PVA hybrid nanofibers. The whole process is made up of three steps: fabrication of Ag nanoparticles, formation of Ag nanoparticles/PVA hybrid solution and preparation of Ag nanoparticles/PVA hybrid nanofibers via an electrospinning technique. In comparison with the procedure described in a previous report [12], the present procedure can avoid reducing the silver ions slowly by the PVA in the PVA/AgNO_3_ hybrid solution before electrospinning (Figure S1), or reducing of the silver ions by a stainless steel needle tip during electrospinning the PVA/AgNO_3_ hybrid solution [13].

**Figure 2:**
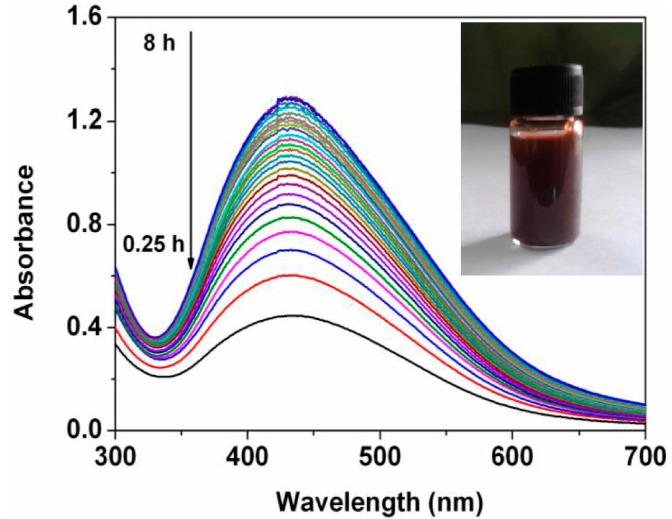
UV-Vis spectra of as-prepared Ag nanoparticles at different reaction time. The insert shows image of the reaction medium.

UV-Vis spectroscopy was employed to confirm formation of the Ag nanoparticles. Figure 2 presents the UV-Vis spectra recorded from the reaction medium at different reaction time. It is observed that there are significant absorption peaks at 428 nm. The absorbance at 428 nm increases with increase of time range from 0.25 h to 8 h. An insert shows color of reaction medium after 7 h of incubation of the extract with the silver nitrate solution at room temperature. The absorbance at 428 nm as well as appearance of the specific color is due to the excitation of surface plasmon resonance which indicates reduction of the silver ions into the Ag nanoparticles [14].

**Figure 3:**
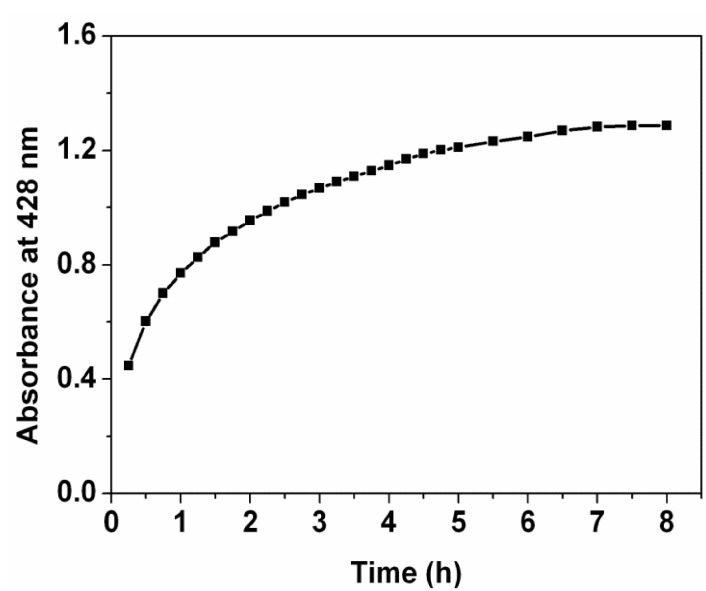
UV-Vis kinetic study of Ag nanoparticles formation.

To understand time-dependent kinetics of the Ag nanoparticles synthesis by the extract at room temperature, a change of absorbance at 428 nm was monitored with time during formation of the Ag nanoparticles. It is found that the absorbance intensity increases abruptly within 0.9 h of reaction time (Figure 3); the reaction is 58.2% complete. After that, it increases slowly up to 7 h; the reaction is 99.7% complete. The optimum time required for the completion of the reaction is recorded to be 7 h. In the following 1 h of the reaction time, it is also found that the absorbance does not change, implying that as-prepared Ag nanoparticles are stable without the help of chemical stabilizers. In previous reports on fabricating Ag nanoparticles/PVA hybrid nanofibers [15], The Ag nanoparticles were synthesized via several methods, such as chemical reduction under heating at 105 °C, chemical reduction within 96 h of reaction time at room temperature, or photoreduction under UV irradiation. In the present study, the Ag nanoparticles were successfully fabricated through the reduction with the *Ficus altissima Blume* leaf extract at room temperature. The present method owns advantages of time-saving, energy-saving and eco-friendly. Therefore, the present study provides a novel and sustainable route to fabricating the Ag nanoparticles/PVA hybrid nanofibers.

**Figure 4:**
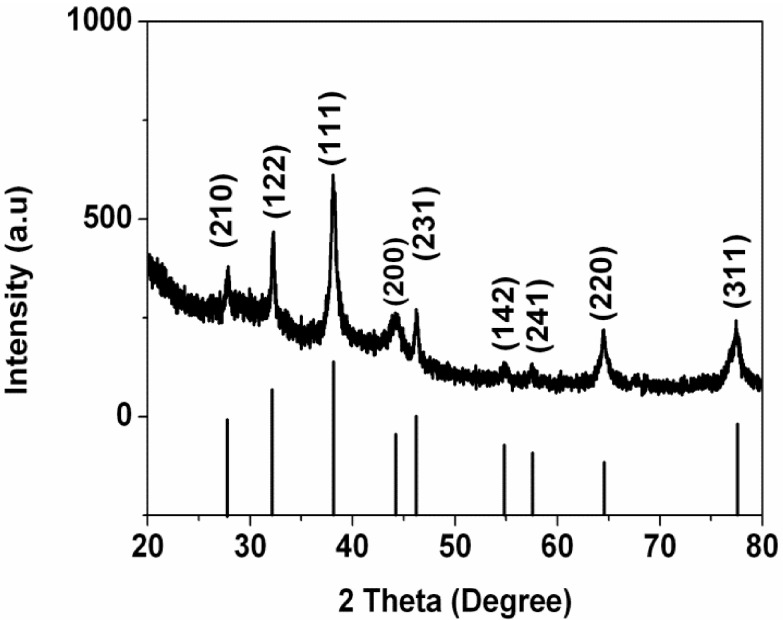
X-ray diffraction (XRD) pattern of obtained Ag nanoparticles.

X-ray diffraction pattern of the formed Ag nanoparticles is shown in Figure 4. A number of Bragg reflection peaks were observed at 2θ values of 27.81°, 32.16°, 38.12°, 44.3°, 46.21°, 54.83°, 57.39°, 64.42° and 77.45° which are indexed to (210), (122), (111), (200), (231), (142), (241), (220) and (311) planes of pure silver based on the face-centered cubic structure (JCPDS, file No. 04-0783) [16,17]. The XRD results clearly show that the Ag nanoparticles synthesized by the extract are crystalline in nature. The size of Ag nanoparticles was calculated by using Debye-Scherer’s equation *D* = 0.9λ/βcosθ, where *D* is the crystalline size, λ is the wavelength of X-ray, β is the full width at half maximum of the diffraction peak and θ is the Bragg’s angle. The size of obtained Ag nanoparticles is estimated around 6.4 nm from the breadth of the (111) reflection.

**Figure 5:**
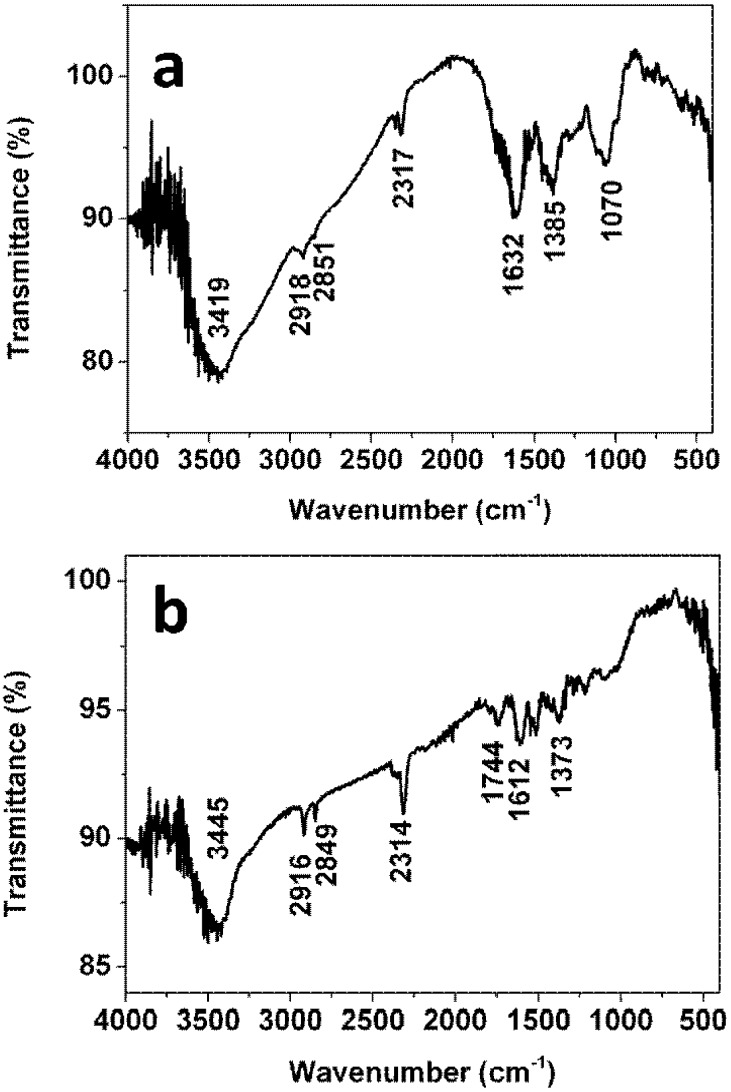
Fourier transform infrared (FTIR) spectra of the extract (**a**) and the obtained Ag nanoparticles (**b**).

Fourier transform infrared spectroscopy of the *Ficus altissima Blume* leaf extract is presented in Figure 5a. Some absorption bands at 3419, 2918, 2851, 2317, 1632, 1385 and 1070 cm^−1^ are observed in the region 1000–4000 cm^−1^. The band at 3419 cm^−1^ is attributed to –OH group [18]. The bands at 2918 cm^−1^ and 2851 cm^−1^ are assigned to –CH_2_ and C–H stretching, respectively. The band at 1632 cm^−1^ corresponds to conjugated –C=C [19]. The band at 1070 cm^−1^ is due to stretching vibration of –C–O which may derives from polyols such as flavones, terpenoids, or polysaccharides in the leaf extract. To compare with the FTIR spectrum of the leaf extract, FTIR spectrum of the obtained Ag nanoparticles is also given in Figure 5b. Several absorption bands located at 3445, 2916, 2849, 1744, 1612 and 1373 cm^−1^ are observed in the region 1000–4000 cm^−1^. Among these absorption bands, the bands at 3445, 2916, 2849, 1612 and 1373 cm^−1^ remain nearly the same while a fresh band appears at 1744 cm^−1^ which is assigned to the stretching vibration of –C=O [20]. In addition, it is also observed that band at 1070 cm^−1^ disappears after formation of the Ag nanoparticles, which is attributed to the stretch vibration of –C–O. Hence, it is suggested that the alcohol group (–C–OH) should be converted to the carbonyl group (–C=O) during the reduction with the silver ions. The adsorption of obtained carbonyl group onto the Ag nanoparticles plays a role in stabilizing Ag nanoparticles. On the basis of results of the above FTIR spectra, the reaction between the extract and the silver ions might occur according to the following equation: Ag^+^ + R–OH → R = O + Ag + H^+^. The decrease of pH of resulting solution further confirms formation of hydrogen ions. The pH at the point when AgNO_3_ is added is 5.79 while the pH of the mixture after reaction is 3.70.

**Figure 6:**
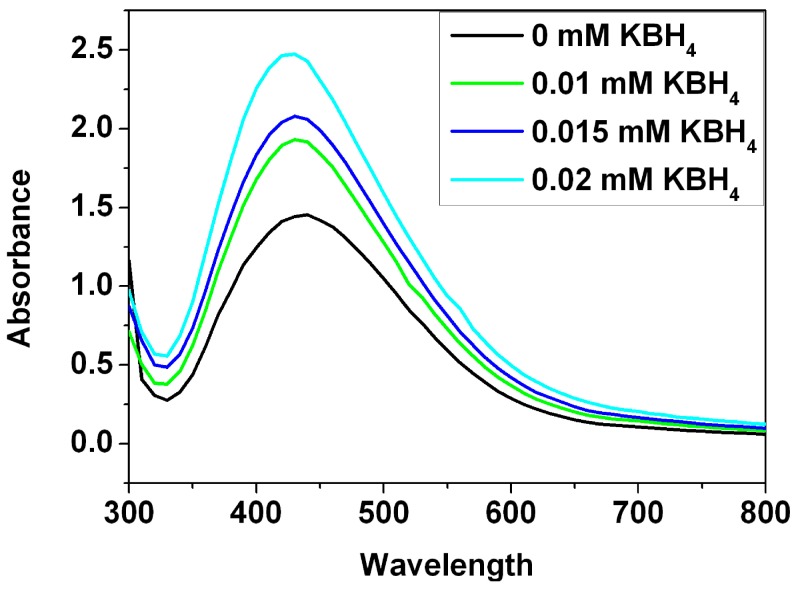
UV-Vis study of the Ag nanoparticle stability as a function of increasing KBH_4_ concentration.

To gain insight into stability of the Ag nanoparticles in the presence of electrolyte, the effect of KBH_4_ (potassium borohydride) on the stability of the Ag nanoparticles was investigated (Figure 6). After addition of different amounts of KBH_4_, it was observed that width of absorbance band increases with the KBH_4_ concentration increasing. An increase in the width of absorbance band is more pronounced at the concentration of 0.02 mM which implies wider size distribution due to aggregation of the Ag nanoparticles in the presence of KBH_4_. On the basis of zeta potential measurement, the zeta potential value of the Ag nanoparticles in the water is −31.6 mV. In the presence of the KBH_4_ with various concentrations, the positively charged potassium ions are adsorbed on the surface of the negatively charged Ag nanoparticles. Reduction in the zeta potential results in subsequent aggregation. As previously reported [21], the Ag nanoparticles synthesized by plant extracts showed good catalytic activity on reduction of the MB in the presence of NaBH_4_. However, the obtained Ag nanoparticles tend to aggregate and become unstable in the presence of electrolyte, which decreases their activities for the catalytic applications.

**Figure 7:**
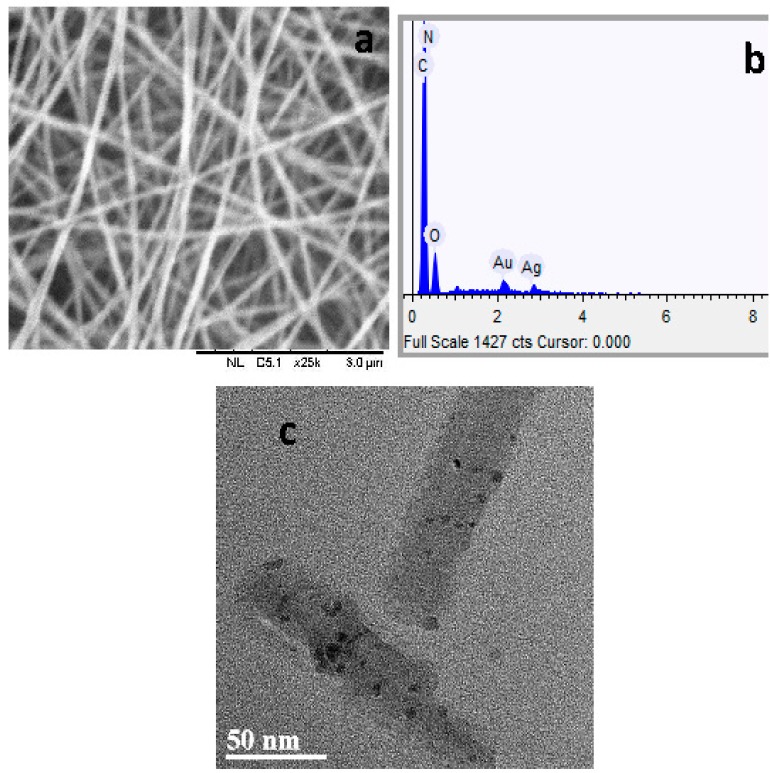
Scanning electron microscopy (SEM) image (**a**); energy dispersive spectrum (EDS) (**b**); transmission electron microscopy (TEM) image (**c**) of prepared Ag nanoparticles/PVA hybrid nanofibers.

To reduce undesirable aggregation of the as-prepared Ag nanoparticles in the presence of KBH_4_, the ecletrospinning technique has been employed to create PVA nanofibers as supports for the Ag nanoparticles. Some morphological characterization of the obtained Ag nanoparticles/PVA hybrid nanofibers was conducted with both SEM and TEM. Some uniform nanofibers with a diameter range from 40 nm to 178 nm are clearly observed in the SEM image (Figure 7a). The energy dispersive spectrum (EDS) of the obtained Ag nanoparticles/PVA hybrid nanofibers is shown in Figure 7b. The result shows the presence of C, N, O and Ag elements with a C/Ag atomic ratio of 73.216/0.018 in the Ag nanoparticles/PVA sample. In addition to those elements, the Figure 7b also presents Au element which was from gold coating spun during preparing sample for the SEM analysis. The size of Ag nanoprticles was determined via the TEM measurement (Figure 7c). It was observed that the Ag nanoparticles are roughly spherical in shape. The average size of Ag nanoparticles is 4.9 nm which is nearly in agreement with the result obtained from Debye-Scherer’s equation.

**Figure 8:**
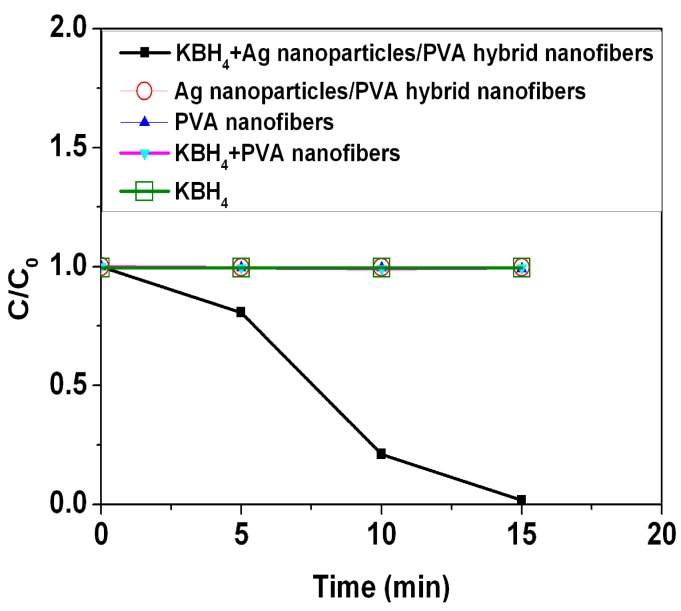
Catalytic effect of Ag nanoparticles/PVA hybrid nanofibers on the reduction of methylene blue (MB).

**Figure 9:**
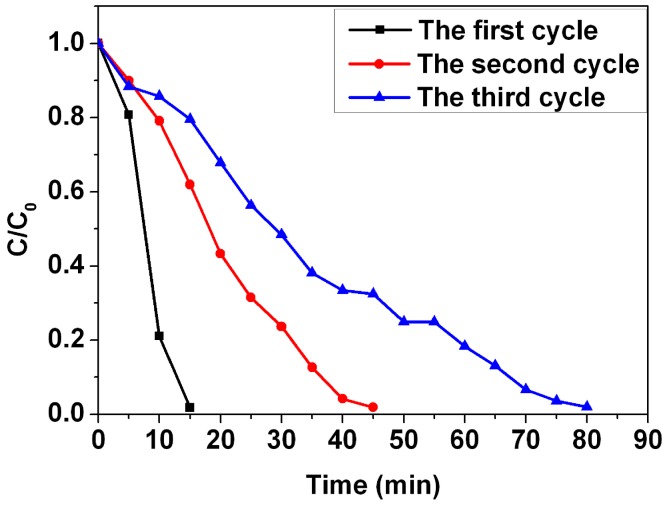
Catalytic kinetics Ag nanoparticles/PVA hybrid nanofibers for three successive reactions of MB.

The catalytic activity of the Ag nanoparticles/PVA hybrid nanofibers was investigated by using reduction of the MB by the KBH_4_. The reduction of the MB to colorless leucomethylene blue (LMB) has been confirmed by a previous report [22]. The maximum absorbance of the MB is located at the wavelength of 665 nm (Figure S2). The catalytic reactions were monitored by using UV-Vis spectroscopy. Figure 8 shows time courses of the relative concentration of MB (*C*/*C*_0_) under several experimental conditions. The results show that addition of the Ag nanoparticles/PVA hybrid nanofibers can produce the fast reduction of the MB in the presence of KBH_4_, the reduction is nearly 100% of the initial concentration after 15 min. In this catalytic process, the Ag nanoparticles/PVA hybrid nanofibers can act as an electron relay system. The Ag nanoparticles start the catalytic reduction by relaying electrons from the donor BH_4_^−^ ions to the acceptor MB molecules where the Ag nanoparticles/PVA hybrid nanofibers accept electrons from BH_4_^−^ ions and conveys them to the MB. Due to the fact that there is no porous network observed in PVA nanofibers in Figure 7c, some entrapped Ag nanoparticles in PVA nanofibers are not involved in the catalytic reduction. In contrast, PVA nanofibers, Ag nanoparticles/PVA hybrid nanofibers, KBH_4_+PVA nanofibers, or KBH_4_ do not show any change in the concentration during the 15 min period. This indicates that the PVA nanofiber is a promising support for the catalytic application of the Ag nanoparticles. The reusability of the Ag nanopaticles/PVA hybrid nanofibers was also investigated for the reduction of the MB (Figure 9). The results demonstrate that 98.2% of the MB has degraded after 15 min in the first cycle, 98.1% of the MB has degraded after 45 min in the second cycle, and 98.1% of the MB has degraded after 80 min in the third cycle. The reduction rate decreases with the catalytic cycle due to the fact that leucomethylene blue, which is generated during the reduction of the MB, is adsorbed on the surface of the catalyst [23]. However, no obvious losses in degradation ratio of MB are observed after three cycles, suggesting that Ag nanoparticles/PVA hybrid nanofibers are stable during the catalytic reaction [24], demonstrating their potential applications in catalysis.

## 3. Experimental Section

### 3.1. Materials

*Ficus altissima Blume* leaves were collected on campus in Hanshan Normal University. AgNO_3_ (Silver nitrate), PVA (polyvinyl alcohol, *M*_w_ = 145,000) and KBH_4_ (potassium borohydride) were purchased from Aladdin Industrial Corporation in Shanghai, China. Distilled water was used throughout the experiments.

### 3.2. Synthesis of the Extract

The leaves were thoroughly washed, dried at room temperature, and chopped into fine slices. 25 g of as-prepared leaves was transferred into a 250 mL round bottom flask containing 100 mL of distilled water, and then boiled for 15 min. The resulting solution was filtered, and extract was obtained. The extract was used for further experiments.

### 3.3. Synthesis of Ag Nanoparticles

Synthesis of Ag nanoparticles was carried out by using the following procedures: 0.1 M aqueous solution of Silver nitrate was prepared and used for the synthesis of silver nanoparticles. 100 mL of distilled water and 2 mL of the extract were taken into 250 mL beaker, and then 2 mL of aqueous solution of silver nitrate was added to form mixture. The mixture was incubated for 7 h at room temperature to obtain the Ag nanoparticles.

### 3.4. Synthesis of Ag Nanoparticles/PVA Hybrid Nanofibers

Without extracting the Ag nanoparticles, PVA was directly dissolved in the obtained Ag nanoparticles solution to prepare 8 wt.% Ag nanoparticles/PVA hybrid solution. The as-prepared Ag nanoparticles/PVA mixture was placed in a plastic syringe fitted with a 21 gauge stainless steel needle tip. A rotating cylinder was used to collect the nanofibers. The applied voltage was 19.6 kV, the solution flow rate was 0.1 mm/min. The distance between the needle tip and the collector was 10 cm. After electrospinning the hybrid solution, the nanofibrous membranes were separated from the cylinder for further characterizations.

### 3.5. Catalytic Experiments

In a typical experiment, 0.078 g of Ag/PVA hybrid nanofibers was immersed into 3.5 mL of mixed solution containing 20 mg/L MB and 10 mM KBH_4_ in a quartz cuvette. The catalytic reduction was monitored by recording the time-dependent absorbance at the wavelength of 665 nm with a UV-Vis spectrophotometer at room temperature.

### 3.6. Characterization

The optical properties were characterized by using UV-Vis spectroscopy (UV–1100, MAPADA INSTRUMENT, Shanghai, China). XRD patterns were recorded using an X-ray diffractometer (Rigaku D/Max 2200PC, Japan Rigaku Corporation, Tokyo, Japan) with a graphite monochromator and CuKα radiation (λ = 0.15418 nm). A TEM (Titan, FEI Company, Hillsboro, OR, USA) and a SEM (TM3030 Tabletop Microscope, Hitachi, Tokyo, Japan) were applied to observe the morphology and microstructure of the samples. Surface image was characterized with FE-SEM (JEOL JSM-6700F, JEOL Japan Electronics Co., Ltd, Tokyo, Japan). The infrared (IR) spectra were measured on a FTIR (Nicolet 5DX, Nicolet, Natus Neurology, Middleton, WI, USA) by using KBr pellet technique.

## 4. Conclusions

The Ag nanoparticles/PVA hybrid nanofibers have been successfully synthesized by a novel and sustainable route. The formed Ag nanoparticles with the average size of 4.9 nm remained in PVA nanofibers demonstrate high stability and reusability during the reduction of MB. The as-prepared Ag nanoparticles/PVA hybrid nanofibers have a potential application in catalytic area.

## Supplementary Materials

Supplementary materials can be accessed at: http://www.mdpi.com/2079-4991/5/2/1124/s1.
