# Supplementary material for: A Sustainable Approach to Fabricating Ag Nanoparticles/PVA Hybrid Nanofiber and Its Catalytic Activity
Source: Nanomaterials (Basel). 2015 Jun 23;5(2):1124–35. doi: 10.3390/nano5021124 (PMC5312901; doi:10.3390/nano5021124)
Supplement: Supplementary file 1 [file nanomaterials-05-01124-s001.pdf]

# Supplementary Information

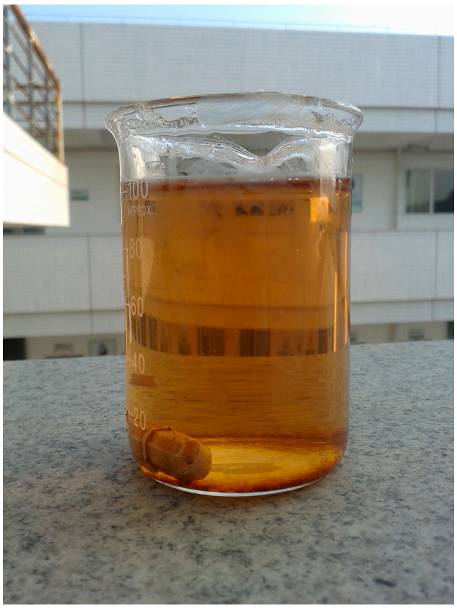

**Figure S1.** Image of $AgNO_3$/PVA hybrid solution before electrospinning.

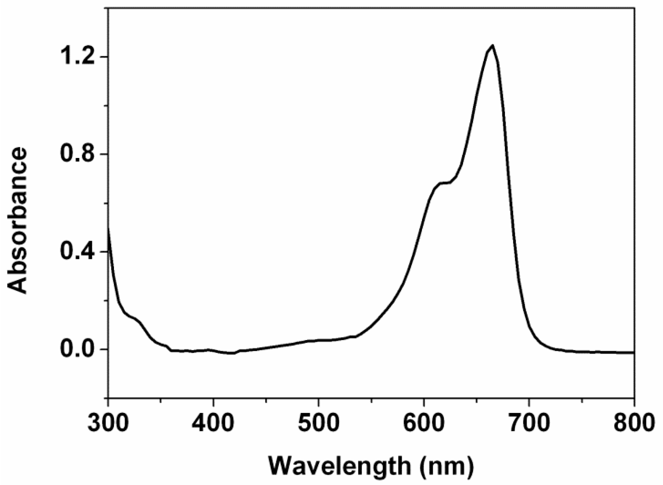


**Figure S2.** UV–Vis spectrum of MB.

© 2015 by the author; licensee MDPI, Basel, Switzerland. This article is an open access article distributed under the terms and conditions of the Creative Commons Attribution license (http://creativecommons.org/licenses/by/4.0/).
